# Dr. Gard Foster

**Published:** 1915-01

**Authors:** 


**Dr. Gard Foster,** Detroit 1878, died at the Ellis Hospital, Schenectady, December 2, 1914, aged 57. He was born June 14, 1857, in Burlington, Vt. After graduation he began practice in Rochester, but moved to Clyde after a few years, and to Auburn in 1905, where he resided until his death, devoting his attention to diseases of the eye, ear, nose and throat, and giving two days each week to charity cases. He had been in the North Woods for some weeks hoping to recover his health, but was removed to Schenectady about two weeks before his death for an operation.

